# Usage and limitations of medical consultation with patients’ families using online video calls: a prospective cohort study

**DOI:** 10.1186/s12911-024-02542-y

**Published:** 2024-05-27

**Authors:** Tetsuro Hayashi, Seiji Bito

**Affiliations:** Division of Clinical Epidemiology, NHO Tokyo Medical Center, 2-5-1 Higashigaoka, Meguro-ku, Tokyo, 152-8902 Japan

**Keywords:** Telehealth, Telecommunications, Telemedicine, Family communication

## Abstract

**Background:**

Few studies have been conducted on the usage of telehealth focusing on consultations between patients’ families and physicians. This study aimed to identify the usage and limitations of online medical consultations with patients’ families compared to the traditional in-person consultations.

**Methods:**

We conducted a prospective cohort study from April 1, 2020, to September 30, 2021, at an educational acute-care hospital in Japan. The study included hospitalized patients aged 20 years or older and their family members for whom an online or in-person medical consultation between the family member and physician was conducted during the hospitalization period. The primary endpoints assessed were three topics pertaining to medical consultation: medical conditions and treatment plans, policies for life-threatening events, and post-discharge support. The secondary endpoint was the number of consultations required.

**Results:**

Online consultations and traditional in-person consultations were provided to 58 and 53 patients’ families, respectively. Of the patients in the online consultation group who underwent multiple consultations, 46 (79%) also underwent in-person consultations. Regarding the topics, all the patients’ families in both consultation groups had consultations on medical conditions and treatment plans; regarding the policy for life-threatening events, 47% of patient families in the online consultation group were consulted compared to 53% of those in the in-person group. Regarding post-discharge support, 59% of patient families in the online group were consulted compared to 40% in the in-person group. In the online consultation group of 58 patients’ families, 188 consultations were conducted, including 95 online and 93 in-person consultations. Consultations on policy for life-threatening events were significantly more frequent in in-person consultations than in online consultations (*p* < 0.05). Regarding post-discharge support, online consultations were significantly more frequent than in-person consultations (*p* < 0.05). The number of family members who attended online consultations was significantly higher than those who attended in-person consultations (*p* < 0.05).

**Conclusions:**

Online consultation between the physician and patient’s family may be an alternative to in-person consultation for explaining medical conditions and treatment plans. However, in-person consultation still plays an important role in sensitive topics, such as policy consultation for life-threatening events.

## Background

The development of digital technology has significantly impacted healthcare. Telemedicine makes it possible to examine and diagnose patients, communicate diagnostic results, and prescribe medical treatments via video calls [1]. Telehealth is a broad concept that encompasses not only telemedicine but also health counseling, disease prevention assistance, and disease education provided by medical professionals [2, 3]. The COVID-19 pandemic has significantly contributed to the expansion of telehealth [4–7].

Electronic and telecommunications technology not only enables telehealth services for patients themselves but can also be a means to assist medical care [8]. During the COVID-19 outbreak, hospitals and nursing homes restricted family visits to patients to prevent infection [9–11]. However, online visits enabled patients to meet their families [12]. In addition, especially in Asia, where patients prefer to make decisions jointly with their families, it is common for family members to be present at the hospital when the physician explains the patient’s medical condition and discusses treatment plans [13, 14]. The involvement of family members in healthcare decision-making is a crucial aspect of patient care. Studies have shown that family involvement can promote a more holistic approach to healthcare that encompasses both medical and emotional support [15, 16]. However, with the recent use of video chats, it is no longer necessary for family members to visit hospitals in person.

Research on telemedicine has been conducted extensively worldwide in recent years. Target diseases for which telemedicine may be useful include psychiatric disorders, cancer, eating disorders, chronic diseases, cardiovascular diseases, and sexually transmitted diseases [4, 17]. However, there have been few studies or reports on the usage of telehealth focusing on consultations between patients’ families and physicians. Therefore, we focused on online consultations with patients’ families and evaluated its usage and limitations by comparing it with traditional in-person consultations.

## Methods

### Design, setting, and participants

This prospective cohort study was conducted from April 1, 2020, to September 30, 2021, at the Department of General Medicine, Tokyo Medical Center, Japan. The Tokyo Medical Center is an educational acute-care hospital with 688 beds, including 28 intensive care units and 48 psychiatric beds.

The study included hospitalized patients aged 20 years and older and their families for whom medical consultation between the patient’s family and physician takes place during the hospitalization period. Medical consultations in this study included the following topics: explanation of medical conditions and treatment plans, policy for life-threatening events, and post-discharge support. Policy consultation for life-threatening events included Do Not Attempt Resuscitation (DNAR) instructions and end-of-life care consultations.

The physicians included patients and their families in the study, providing informed consent through explanations, accompanied by a written consent form. The method of data collection involved chart review from electronic medical records, which was conducted after the patients’ discharge. The data collection was conducted by research staff who were separate from those responsible for data analysis.

### Exposure and outcome variables

Medical consultations were provided to the patients and their families who consented to the study. If the family agreed to a videoconference, an online medical consultation was provided; if they did not agree, a traditional in-person medical consultation at the hospital was provided. In cases where multiple consultations were scheduled, families in the online consultation group were allowed in-person consultations at their request. When conducting online consultations, we ensured the same level of privacy as in-person consultations at hospitals, in accordance with the “Guidelines for the Appropriate Implementation of Online Medical Services” set forth by the Japanese Ministry of Health, Labour and Welfare [18]. The primary endpoints assessed were three topics pertaining to medical consultation: medical conditions and treatment plans, policies for life-threatening events, and post-discharge support. The secondary endpoint was the number of consultations during the hospital stay.

### Statistical methods

The Wilcoxon rank-sum test was used to analyze continuous variables, and the chi-square or Fisher’s exact test was employed for categorical variables, depending on appropriateness for data distribution and sample size. The significance was set at *p* < 0.05. All analyses were conducted using the STATA 17 software (StataCorp, College Station, TX, USA).

## Results

Consent for participation in the study was obtained from 117 patients and their families. Of these, 111 patients and their families who received either online or in-person medical consultations during their hospital stay were included in the study (Table 1). Online consultations were provided to 58 patients’ families, and traditional in-person consultations were provided to 53 patients’ families. The mean (standard deviation, ±SD) age of eligible patients was 82.6 (± 13.1) years. Regarding living conditions prior to admission, 77 (69%) patients were living at home and 63 (57%) patients were living with family members.

**Table 1:** Comparison of patient characteristics between the online and in-person family consultation groups

		Online consultation group	In-person consultation group	*p*-value
		(*n* = 58)	(*n* = 53)	
Age (years)		83.3 ± 11.4	81.9 ± 14.8	0.89
Female		30 (52)	26 (49)	0.78
Residence				< 0.05
	Home	34 (59)	43 (81)	
	Other than home	24 (41)	10 (19)	
Living with family		24 (42)	39 (74)	< 0.05
Diagnosis				0.39
	Infectious diseases	27 (47)	21 (40)	
	Stroke/neurological diseases	18 (31)	13 (25)	
	Malignancy	2 (3)	5 (9)	
	Others	10 (17)	12 (23)	

Data are presented as number (%) except for age, which is presented as mean ± SD

The outcome measures are presented in Table 2. The mean (± SD) number of meetings was 3.2 (± 1.7) times in the online consultation group, while 2.7 (± 2.0) times in the in-person consultation group. Of the patients in the online consultation group who underwent multiple consultations with their family members, 46 (79%) also underwent in-person consultations. Regarding the topics, all patients’ families in both the online and in-person consultation groups had consultations on medical conditions and treatment plans. Regarding the policy for life-threatening events, 47% of patients’ families in the online consultation group were consulted compared to 53% of those in the in-person group. Regarding post-discharge support, 59% of patients’ families in the online group were consulted compared to 40% in the in-person group.

**Table 2:** Outcome measures

		Online consultation group	In-person consultation group
		(*n* = 58)	(*n* = 53)
Meeting (number of times)			
	1	8 (14)	15 (28)
	2	13 (22)	16 (30)
	3	18 (31)	11 (21)
	4	8 (14)	7 (13)
	>=5	11 (19)	4 (8)
Consultation			
	In-person	46 (79)	53 (100)
	Online	58 (100)	0 (0)
Topics of consultation			
	Medical condition and treatment plan	58 (100)	53 (100)
	Policy for life-threatening event	27 (47)	28 (53)
	Post-discharge support	34 (59)	21 (40)
Number of family members		1.7 ± 0.8	1.5 ± 0.5
Hospital stay (days)		32.6 ± 25.5	27.7 ± 25.0
Discharge disposition			
	Home	19 (33)	24 (45)
	Nursing facilities	16 (28)	5 (9)
	Other hospitals	18 (31)	12 (23)
Death during hospitalization		5 (9)	12 (23)

Data are presented as number (%) except for number of family members and hospital stay, which are presented as mean ± S.D.

The topics of all consultations provided to the patients’ families in the online consultation group are shown in Table 3. A total of 188 consultations were conducted with 58 patients’ families: 95 online and 93 in-person. Consultations on policy for life-threatening events were significantly more frequent during in-person consultations (*p* < 0.05). Post-discharge support was significantly more frequent during online consultations than during in-person consultations (*p* < 0.05). The number of family members who attended online consultations was significantly higher compared to those who attended in-person consultations (*p* < 0.05).

**Table 3:** Analysis of all consultations for 58 patients in the online consultation group

		Online consultation	In-person consultation	*p*-value
Number of consultations		95	93	
Topics of consultation				
	Medical condition and treatment plan	94 (99)	91 (98)	0.62
	Policy for life-threatening event	6 (6)	27 (29)	< 0.05
	Post-discharge support	28 (29)	11 (12)	< 0.05
Number of family members		2.0 ± 1.1	1.6 ± 0.7	< 0.05

Data are presented as number (%) except for number of family members, which is presented as mean ± S.D

## Discussion

This study aimed to assess the usage and limitations of online consultations with patients’ families in comparison to traditional in-person consultations. The timing of the study coincided with the COVID-19 pandemic, which limited family visitations and opportunities for in-person family-physician consultations [9–11]. Under these circumstances, approximately half of the patients’ families agreed to incorporate online consultations. These family members consulted physicians to share medical information and reach a consensus on treatment and care plans through online consultations.

The study analyzed the consultation methods, number of consultations, and topics between the group that received online consultations and the group that received only in-person consultations. Only 21% of the patients’ family members who agreed to participate in online consultations completed all consultations online. This suggests that online consultation is currently used as a complementary tool in the hospital medical consultation process and has not yet reached the point where it can replace traditional in-person consultations. However, in terms of the number of consultations, more family members in the online consultation group had three or more consultations during their hospital stay than those in the in-person consultation group. This suggests that the addition of an online option for family physician consultation may improve accessibility for the patient’s family. The high number of family members participating in online consultations may provide evidence of improved access. Nevertheless, without proportionate analysis, raw numbers might not fully capture the nuances of improved access. The higher mortality among patients who had in-person consultations hints at a preference for direct consultations among the families of critically ill patients; this suggests the need for more personal interaction in severe cases.

In terms of consultation topics, those explaining the medical conditions and treatment plans were conducted for all patients in both groups. In the online group, the same level of medical conditions and treatment plan explanations were provided as in the in-person group. These results suggest that online consultations are an alternative to in-person consultations for sharing medical information and reaching consensus on treatment plans. However, differences between in-person and online consultations were observed, even within the online consultation group, regarding the topics of policy for life-threatening events and post-discharge support.

Understanding the appropriate role of family communication in end-of-life care decisions is important to ensure that patients receive timely and appropriate medical care at the end of life [13]. Consultations on policies for life-threatening events are a high priority in Japan, where the resuscitation rate is inevitably low due to the large number of hospitalized older adult patients [19]. The Ministry of Health, Labour and Welfare has also established “Guidelines for the Decision-Making Process of Medical Care in end-of-life care,” and the role of family members as proxies for decision-making is especially important for patients who lack decision-making capacity [20]. Policymaking regarding life-threatening events tended to be discussed more during in-person consultations than online. One factor may be the psychological barrier on the physicians’ side to withholding medical treatment, making it difficult to discuss sensitive topics directly related to a patient’s life online.

Post-discharge support consultations were conducted more frequently in the online group as many patients in the online group had to shift their care to a place other than home because of family or medical conditions. Additionally, post-discharge support consultations were conducted more frequently online than during in-person consultations, even within the online group. Considering that more family members attended online consultations than in-person consultations, online consultations may have been more convenient for post-discharge care support consultations.

Our study had several limitations that must be considered. First, this was a prospective cohort study based on family preferences rather than a randomized controlled trial. However, the study period was during the COVID-19 pandemic, and we were convinced that providing a consultation environment based on family preferences was a priority. Second, even in the online group, we adopted a hybrid format in which not all consultations were practiced online, but in-person consultations were also conducted depending on the patient’s situation and family requests. However, an analysis of the topics of consultations in the online group suggested that consultations directly related to life-threatening issues may be preferably conducted in person because of their sensitivity.

This study was conducted in Japan, which is characterized by its aging society, where family involvement in healthcare decision-making plays a pivotal role, especially for the older adult patient demographic. This context demonstrates the importance of family members in supporting patient decisions, particularly when restrictions prevent their physical presence at the hospital. The utility and convenience of online consultations has become markedly evident in such scenarios, offering a practical solution for remote family involvement. The outcomes of this study are particularly relevant for countries on the cusp of becoming aging societies and for those with families living far apart.

Future research should focus on conducting qualitative studies to delve deeper into the reasons underlying the study findings, such as investigating the quality of support and family satisfaction with online versus in-person consultations. Additionally, further studies should examine how the severity of a patient’s condition affects their family’s preference of consultation method and the outcomes of these consultations.

Currently, online consultations serve as a valuable complement to traditional in-person consultations, facilitating enhanced communication between healthcare providers and patients’ families. However, based on the evidence from our study, it will be premature to conclude that online consultations can fully replace in-person interactions. This is particularly true in the context of decision-making, especially when dealing with actions directly related to life, such as policies for life-threatening events, is delicate, and at present, physicians may not feel confident of consulting online unless they directly observe the expressions and reactions of patients and their families. If the problem is only a psychological barrier on the part of the physician, it can be overcome through specialized training. However, the family members of patients who receive explanations may also have barriers to receiving sensitive information online. Hence, the society needs to reach a more mature stage in which it is accustomed to online communication. At present, it is necessary for medical professionals to share a common understanding of which agendas can be complemented online and which are preferable in person, and to make good use of online consultations. With little research conducted in this area, it is expected to become an important area of research in the future.

## Conclusion

In conclusion, this study shows that online consultation between physicians and patients’ families may be an alternative to in-person consultation for explaining medical conditions and treatment strategies. However, in-person consultations still play an important role in sensitive consultations, such as policy consultations for life-threatening events.

## Data Availability

The datasets used and/or analyzed during the current study are available from the corresponding author on reasonable request.

## Abbreviations

DNAR: do not attempt resuscitation
SD: standard deviation
